# HIV Exploits Antiviral Host Innate GCN2-ATF4 Signaling for Establishing Viral Replication Early in Infection

**DOI:** 10.1128/mBio.01518-16

**Published:** 2017-05-02

**Authors:** Guochun Jiang, Clarissa Santos Rocha, Lauren A. Hirao, Erica A. Mendes, Yuyang Tang, George R. Thompson, Joseph K. Wong, Satya Dandekar

**Affiliations:** a Department of Medical Microbiology and Immunology, University of California, Davis, California, USA; b Department of Molecular and Cellular Biology, University of California, Davis, California, USA; c Department of Medicine, University of California, San Francisco, and San Francisco VAMC, San Francisco, California, USA; Texas Biomedical Research Institute; Albert Einstein College of Medicine

**Keywords:** ATF4, GCN2, HIV latency, HIV replication, HIV

## Abstract

Antiviral innate host defenses against acute viral infections include suppression of host protein synthesis to restrict viral protein production. Less is known about mechanisms by which viral pathogens subvert host antiviral innate responses for establishing their replication and dissemination. We investigated early innate defense against human immunodeficiency virus (HIV) infection and viral evasion by utilizing human CD4^+^ T cell cultures *in vitro* and a simian immunodeficiency virus (SIV) model of AIDS *in vivo*. Our data showed that early host innate defense against the viral infection involves GCN2-ATF4 signaling-mediated suppression of global protein synthesis, which is exploited by the virus for supporting its own replication during early viral infection and dissemination in the gut mucosa. Suppression of protein synthesis and induction of protein kinase GCN2-ATF4 signaling were detected in the gut during acute SIV infection. These changes diminished during chronic viral infection. HIV replication induced by serum deprivation in CD4^+^ T cells was linked to the induction of ATF4 that was recruited to the HIV long terminal repeat (LTR) to promote viral transcription. Experimental inhibition of GCN2-ATF4 signaling either by a specific inhibitor or by amino acid supplementation suppressed the induction of HIV expression. Enhancing ATF4 expression through selenium administration resulted in reactivation of latent HIV *in vitro* as well as *ex vivo* in the primary CD4^+^ T cells isolated from patients receiving suppressive antiretroviral therapy (ART). In summary, HIV/SIV exploits the early host antiviral response through GCN2-ATF4 signaling by utilizing ATF4 for activating the viral LTR transcription to establish initial viral replication and is a potential target for HIV prevention and therapy.

## IMPORTANCE

Understanding how HIV overcomes host antiviral innate defense response in order to establish infection and dissemination is critical for developing prevention and treatment strategies. Most investigations focused on the viral pathogenic mechanisms leading to immune dysfunction following robust viral infection and dissemination. Less is known about mechanisms that enable HIV to establish its presence despite rapid onset of host antiviral innate response. Our novel findings provide insights into the viral strategy that hijacks the host innate response of the suppression of protein biosynthesis to restrict the virus production. The virus leverages transcription factor ATF4 expression during the GCN2-ATF4 signaling response and utilizes it to activate viral transcription through the LTR to support viral transcription and production in both HIV and SIV infections. This unique viral strategy is exploiting the innate response and is distinct from the mechanisms of immune dysfunction after the critical mass of viral loads is generated.

## INTRODUCTION

Acute viral infections trigger an antiviral host innate defense response in the infected host for restricting viral replication and dissemination (1). However, viral pathogens have evolved mechanisms either to overcome host antiviral mechanisms or to exploit them for supporting viral replication. Human immunodeficiency virus type 1 (HIV) establishes persistent infection in humans and is the cause of the AIDS epidemic worldwide (2). Gut-associated lymphoid tissue is an early target organ for the establishment of HIV infection and dissemination and a site for viral persistence (3). The impact of established chronic HIV infection in therapy-naive individuals is well documented by the incidence of gastrointestinal complications, including nutrient malabsorption, impaired immunity, and metabolic dysfunction. The role of infectious virus and viral antigens in the pathogenesis of immune dysfunction in HIV infection has been well investigated (4–7). However, our understanding of the early host-virus interactions at the mucosal sites is limited. During the initial encounter with the susceptible host cell targets, HIV infection triggers a signaling cascade that induces multiple innate antiviral defenses and primes the host for generating virus-specific immune responses (8). Mechanisms by which HIV leverages some of the host innate defenses for its own benefit are not fully explored. This feature is especially critical for the virus to invade and establish infection in the gut mucosa since the gastrointestinal tract is immunologically highly evolved to combat the incoming pathogens and is richly endowed with innate immune defenses (9). We propose that the virus might utilize the early antiviral host innate response for supporting its own transcription/replication and to build a critical mass of viral loads at the site of infection. This step is critical for advancing viral dissemination to other susceptible targets and for impairing host immunity.

HIV is greatly dependent on the host cell protein synthetic machinery for producing viral proteins and infectious viral particles. It has been well established that acute viral infections activate an antiviral response in host cells that includes inhibition of protein synthesis and amino acid deprivation (10). This leads to the restriction of the viral protein production and results in the control of the viral infection through the mechanism of phosphorylation of eukaryotic translation initiation factor 2 alpha (eIF2α). HIV infection of cell lines *in vitro* induced inhibition of protein translation and increased expression of the protein kinase general control nonderepressible 2 (GCN2) (10). Phosphorylation of eIF2α by activated GCN2 converts eIF2 to a competitive inhibitor of eIF2B and results in the inhibition of protein synthesis (11). Despite the suppression of global protein synthesis during viral infections, a specific set of mRNAs is preferentially translated, including the transcription factor ATF4 (12). It is not known whether ATF4 expression is altered during early stages of HIV/simian immunodeficiency virus (SIV) infections and whether it activates HIV transcription. Therefore, we sought to investigate the host antiviral response integrating GCN2 activation and amino acid deficiency-driven ATF4 expression to understand whether the virus would exploit these mechanisms for enhancing its own replication and expansion. The SIV model of AIDS has been valuable for unraveling the mechanisms of HIV pathogenesis. A combination of the SIV model and well-established HIV cell culture systems *in vitro* provides an excellent opportunity to interrogate early stages of viral infection in the context of host antiviral innate defense and mechanisms of viral evasion or exploitation of the host antiviral pathways for establishing early viral reservoirs (10, 13–16).

In this study, our data show rapid suppression of protein biosynthesis and amino acid metabolism in the gut mucosa of rhesus macaques during very early stages of SIV infection *in vivo* (within 60 h of infection). This effect was linked to activation of GCN2-ATF4 signaling in the gut mucosa. These data were validated by the induction of ATF4 gene expression in primary human CD4^+^ T cells following direct HIV infection *ex vivo*. Experimentally induced amino acid deficiency in CD4^+^ T cell cultures *in vitro* resulted in the induction of ATF4 expression, which supported increased HIV transcription. ATF4 binding to the HIV long terminal repeat (LTR) was detected during amino acid deficiency. In the CD4^+^ T cells under nutrient insufficiency conditions, addition of GCN2-specific inhibitor or supplementation of amino acids to the cells resulted in the suppression of HIV reactivation. We propose that the viral infection-induced host response of amino acid deficiency induces ATF4 expression. ATF4 in turn binds to the viral promoter in the LTR region and promotes HIV replication. Our findings suggest that while GCN2-ATF4 signaling is activated as a host antiviral innate defense to limit viral protein translation and infection, HIV/SIV is able to hijack GCN2-ATF4 signaling for its own replication and establishment of infection. The HIV infection-induced ATF4 signaling pathway could be targeted as a new strategy to confront latent HIV infection.

## RESULTS

### *In vivo* metabolic changes in the gut mucosa during early SIV infection.

To determine the early host antiviral response to SIV infection, intestinal tissue and peripheral blood samples were obtained from rhesus macaques at 60 h (early stage of infection) and 10 weeks (chronic stage of viral infection) following SIV infection. Progression of infection was monitored by the measurement of viral loads and CD4^+^ T cell numbers (Table 1). Effects of the viral infection on the host were examined through metabolic analysis. During early SIV infection, plasma viral loads ranged from 301 to 1,106 SIV RNA copies/ml while the gut tissue viral loads ranged from 86 to 562 SIV copies/µg total RNA (*n* = 3). No detectable loss of CD4^+^ T cells was observed in the peripheral blood and intestine of animals at 60 h following SIV infection (17). The CD4^+^ T cell numbers in peripheral blood were similar to preinfection values (average, 1,002 cells/μl), while CD4^+^ T cell percentages in the gut ranged between 41 and 53% and were similar to those of uninfected controls (*n =* 4). In contrast, loss of CD4^+^ T cells in both peripheral blood and gut mucosa was detected during chronic SIV infection. Plasma viral loads ranged from 2.3 × 10^4^ to 2.4 × 10^5^ RNA copies/ml, while viral loads in intestinal tissue ranged from 5.7 × 10^3^ to 2.4 × 10^5^ SIV copies/μg total RNA (again, *n =* 4). The CD4^+^ T cell numbers in the peripheral blood ranged from 217 to 1,178 cells/μl (605 ± 407 cells/μl) at 10 weeks post-SIV infection compared to preinfection values of 1,009 to 1,792 cells/ml (1,269 ± 355 cells/μl), while CD4^+^ T cell percentages in the gut were substantially lower than in the controls, suggesting the occurrence of CD4^+^ T cell loss that is the hallmark of progressive SIV infection.

**TABLE 1:** Animals for metabolic analysis^a^

Animal ID	SIV status	Wk postinfection	Viral load (SIV copies/ml plasma)	Gut viral load (SIV copies/μg RNA)	Gut CD3^+^ CD4^+^ T cells (%)
39114	Negative	0	NA	NA	54.3
37301	Negative	0	NA	NA	51.6
37467	Negative	0	NA	NA	70.4
39343	Negative	0	NA	NA	61
35875	Positive	2.5	1,106	114	53.8
36429	Positive	2.5	301	301	53.6
36097	Positive	2.5	263	86	56.6
39296	Positive	10	79,738	566,710	1.0
36701	Positive	10	49,036	18,865	0.2
36719	Positive	10	23,291	13,513	25.9
39238	Positive	10	242,970	11,526	6.4

^a^ Abbreviations: ID, identifier; NA, not applicable.

**FIG 1:**
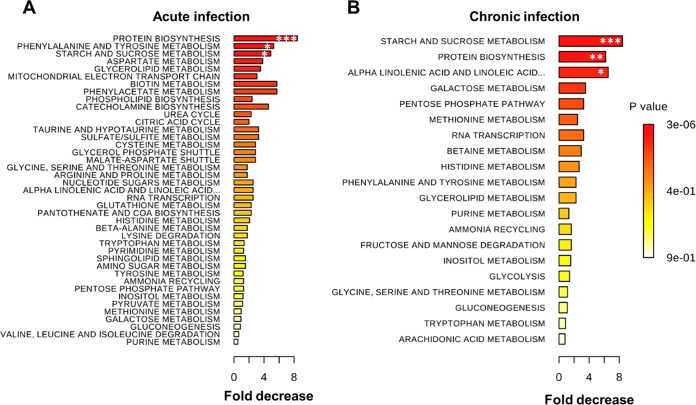
Metabolic changes and amino acid deficiency in the gut during early SIV infection. Top metabolic pathways downregulated in the gut of rhesus macaques after 60 h (A) and at 10 weeks (B) post-SIV infection. ***, *P* < 0.001; **, *P* < 0.01; *, *P* < 0.05 compared with samples from uninfected animals.

To examine the impact of SIV infection on the metabolism in the gut *in vivo*, metabolomic analysis of intestinal samples from SIV-infected animals compared to uninfected controls was performed. Data showed that several metabolic pathways were downmodulated at 60 h of SIV infection compared to SIV-negative controls (Fig. 1A). Changes in protein synthesis and amino acid metabolism processes were particularly notable, as multiple amino acids (phenylalanine, tyrosine, and aspartate and, to a lesser extent, cysteine, glycine, serine, threonine, arginine, proline, histidine, beta-alanine, lysine, tryptophan, methionine, valine, leucine, and isoleucine) and their metabolism were inhibited. However, downmodulation of protein synthesis and amino acid metabolism was less pronounced while starch and sucrose metabolism was significantly downregulated during chronic SIV infection (Fig. 1B). Our findings suggest that downmodulation of protein synthesis and amino acid metabolism occurred early in SIV infection.

### Rapid induction of ATF4 gene expression *in vivo* during early SIV infection but not during chronic SIV infection.

**FIG 2:**
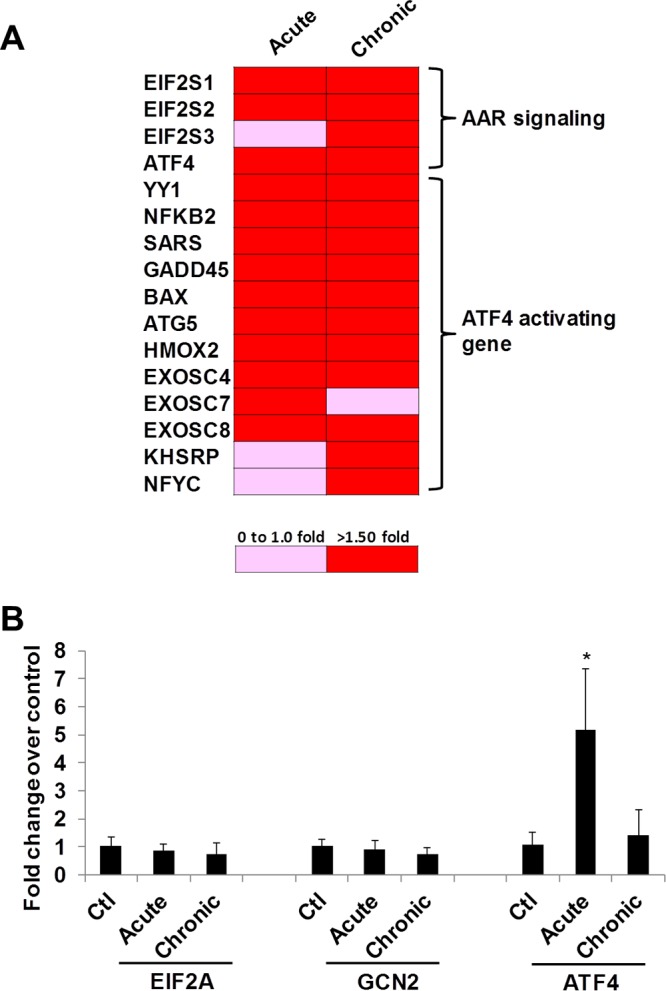
Gene expression of ATF4 is induced in the gut during early SIV infection. (A) ATF4 signaling was activated in gut tissues of rhesus macaques during SIV infection, AAR, amino acid response. (B) Intestinal tissues from acute or chronic SIV-infected rhesus monkeys were collected, RNA was extracted, and expression of GCN2, eIF2α, or ATF4 was determined by RT-qPCR. *, *P* < 0.05.

**FIG 3:**
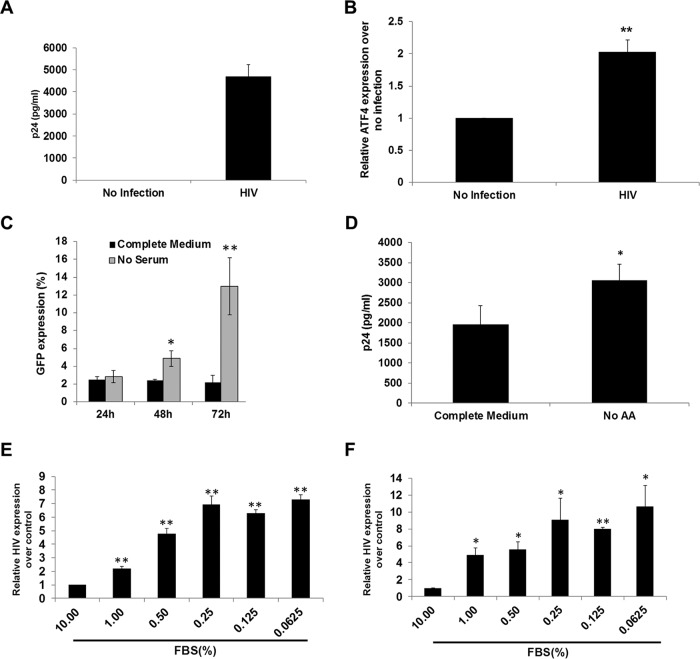
Nutrient deprivation in human CD4^+^ T cells induces ATF4 expression and HIV LTR activation. (A and B) Isolated primary CD4^+^ T cells from healthy HIV-negative donors (*n* = 4) were infected with HIV for 5 days, HIV p24 levels in supernatants were measured by ELISA (A), and cell-associated ATF4 RNA levels were determined by RT-qPCR (B). (C) To determine the effect of nutrient deprivation, J-Lat A1 cells were cultured in medium without serum for 24 to 72 h, and HIV LTR-driven GFP expression was assessed by flow cytometry. (D) Primary human CD4^+^ T cells were infected with HIV in complete medium or in medium without amino acids, and HIV p24 expression was determined by ELISA. (E) J-Lat A1 cells were cultured in low levels of serum (10% to 0.0625% of serum in medium) for 48 h, and HIV LTR-driven GFP expression was measured by RT-qPCR. (F) The U1 cells were cultured in low levels of serum (10% to 0.0625% of serum in medium) for 48 h. HIV RNA was measured using RT-qPCR. *, *P* < 0.05; **, *P* < 0.01. Experiments were performed a minimum of three times.

Metabolic dysfunction and amino acid deficiency are reported in HIV-infected patients and in SIV-infected nonhuman primates (13–15). The amino acid deficiency sensor GCN2-ATF4 signaling directs modulation of transcription control and protein synthesis (12). To determine the role of ATF4 signaling during early SIV infection *in vivo*, we examined changes in the expression of ATF4 and the associated gene network in the gut mucosa during primary acute (2 weeks postinfection) and chronic (26 weeks postinfection) stages of SIV infection. We utilized gut mucosal gene expression data using DNA microarray analysis from our previously reported studies and mined the data to investigate changes associated with ATF4 signaling (17). This analysis showed that the expression of ATF4 as well as the genes downstream of the GCN2-ATF4 signaling was increased in SIV-infected animals compared to uninfected controls (Fig. 2A) (18). For the validation of the DNA microarray-based gene expression data, we performed real-time PCR analysis using rhesus macaque gene-specific primers and determined the expression levels of GCN2, eIF2α, and ATF4 genes in the gut tissues of SIV-infected animals and negative controls. It was important to note that ATF4 expression in the gut mucosa was significantly increased during acute SIV infection (1 to 2 weeks postinfection) but not during chronic SIV infection (27 to 28 weeks postinfection) (Table 2) (Fig. 2B). The transcript levels of GCN2 or eIF2α were not significantly altered. The changes might have occurred at the level of protein synthesis or posttranslational modification. To further examine the changes in ATF4 expression in response to early viral infection, we expanded our analysis to *in vitro* primary human CD4^+^ T cells following HIV infection and measured the induction of ATF4 expression. A productive HIV infection of CD4^+^ T cells was established (Fig. 3A). These cells showed induction of ATF4 RNA expression (Fig. 3B), indicating that HIV infection resulted in upregulation of expression of the ATF4 gene. This is consistent with our data from the SIV model showing an increased ATF4 expression *in vivo* during early SIV infection (Fig. 2A). In summary, our data suggest that SIV infection results in host antiviral mechanisms of amino acid deficiency, which induces expression of ATF4.

**TABLE 2:** Animals for gene expression and qPCR analysis^a^

Animal ID	SIV	Wk postinfection	Viral load (copies)/μg RNA
33675	Negative	0	NA
33842	Negative	0	NA
34249	Negative	0	NA
33832	Negative	0	NA
25317	Positive	1	1,290
25462	Positive	1	107
24882	Positive	2	247,931
25363	Positive	2	934
25365	Positive	2	4,484,518
30085	Positive	26	24,322
34785	Positive	27	2,261
34686	Positive	27	125
34829	Positive	27	20,649
33804	Positive	27	35,686
31353	Positive	28	164,544

^a^ Abbreviations: ID, identifier; NA, not applicable.

### Nutrient deprivation induces activation of HIV LTR-driven transcription in CD4^+^ T cell culture model *in vitro*.

To assess whether the antiviral host response of nutrient deprivation and reduced protein biosynthesis has any beneficial effect on HIV replication, we utilized an established *in vitro* HIV latency cell culture model, the J-Lat A1 (J-Lat) cells, which contain one integrated copy of HIV LTR that controls expression of the green fluorescent protein (GFP) reporter gene. The expression of GFP is induced in these cells following the activation of HIV LTR. The J-Lat cells were serum starved and analyzed for the expression of GFP by flow cytometry. Nutrient deprivation of J-Lat A1 cells activated transcription of HIV LTR-directed GFP (Fig. 3C). To examine whether depletion of amino acids modulates HIV replication, primary human CD4^+^ T cells from healthy donors were isolated and experimentally infected with HIV. These cells were incubated in the amino acid-deficient medium, and HIV p24 protein levels in culture supernatants were determined. We found that deprivation of amino acids enhanced HIV replication in the primary CD4^+^ T cells (Fig. 3D). This finding was further confirmed by the detection of increased levels of GFP RNA levels in J-Lat A1 cells as measured by quantitative PCR (qPCR) assays (Fig. 3E). In addition, the amino acid limitation in chronically HIV-infected U1 (a subclone of the human U937 monocytic cell line that is infected with HIV-1) cell cultures *in vitro* resulted in the induction of HIV transcription (Fig. 3F). Our data showed that nutrient deprivation induces HIV transcription and viral replication in both CD4^+^ T cells and monocytes (Fig. 3E and F).

### Nutrient deprivation induces ATF4 transcription in T cells and monocytes *in vitro*.

**FIG 4:**
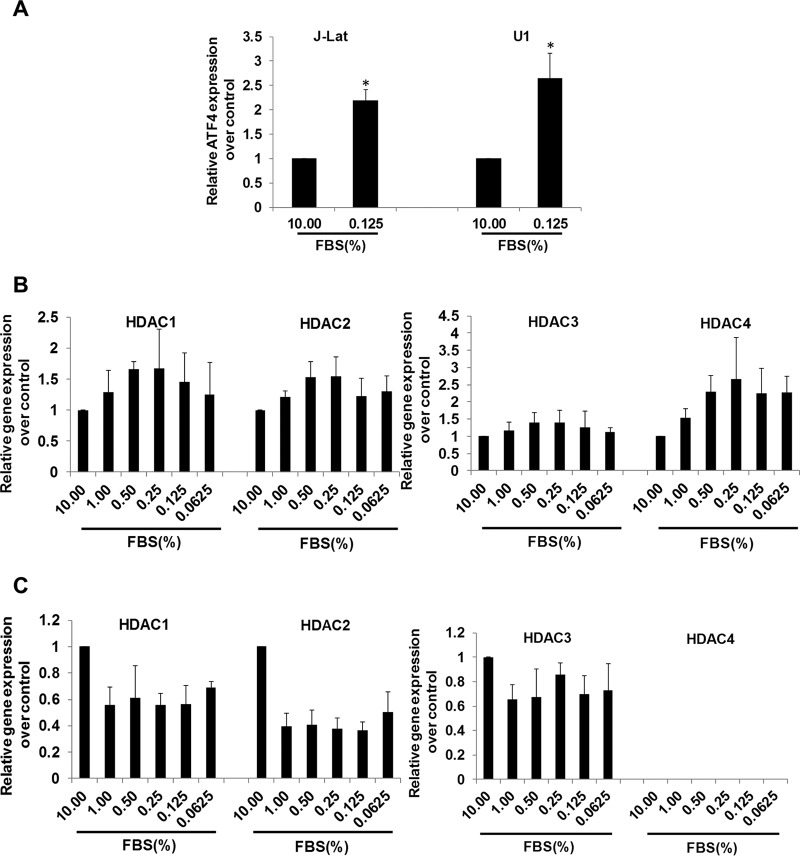
Nutrient deficiency induces ATF4 expression in immune cells. (A) ATF4 expression was induced after nutrient deprivation in both J-Lat A1 cells and U1 cells. J-Lat A1 cells or U1 cells were nutrient deprived for 48 h, and ATF4 expression was evaluated by RT-qPCR. *, *P* < 0.05; **, *P* < 0.01. (B and C) The J-Lat A1 cells (B) and U1 cells (C) were nutrient deprived in medium with low levels of serum (10% to 0.0625%) for 48 h, and HDAC1 to HDAC4 gene expression was evaluated by RT-qPCR. Experiments were performed a minimum of three times.

We sought to examine whether induction of HIV transcription following amino acid deprivation involves changes in the ATF4 expression. We found that ATF4 mRNA expression was induced under nutrient deprivation conditions in both J-Lat cells and U1 cells (Fig. 4A). This is in accordance with our findings of the induction of ATF4 during early SIV infection *in vivo* and in induction of HIV expression *ex vivo* (Fig. 2). It has been previously reported that overexpression of ATF4 can activate HIV expression in CD4^+^ T cells (19). The GCN2-ATF4 signaling is essential in sensing the amino acid deprivation. In both J-Lat A1 and U1 cell culture models, expression of ATF4 was induced under a nutrient-deprived condition. In summary, amino acid deprivation induced HIV expression, possibly through ATF4 signaling.

It was reported that amino acid starvation reactivated latent HIV expression *in vitro*, through downregulation of histone deacetylase 4 (HDAC4) (20). We sought to examine whether induction of HIV transcription following amino acid deprivation also involved changes in the HDAC expression. We analyzed J-Lat A1 cells in the nutrient starvation state for the expression of several HDACs, including HDAC1, HDAC2, HDAC3, and HDAC4. Our data showed that there was no demonstrable suppression of HDAC expression, including that of HDAC4. Instead, expression of most HDACs was induced in J-Lat A1 cells under a serum-starved condition (Fig. 4B). Therefore, nutrient deprivation-induced HIV transcription was not associated with downmodulation of HDACs in J-Lat A1 cells. To determine whether HDAC levels are altered by nutrient deprivation in monocytes, studies were performed in the U1 monocytic cell line that harbors the latent HIV genome. In contrast to J-Lat A1 cells, expression of HDAC1, HDAC2, and HDAC3 was dampened in U1 cells (Fig. 4C). However, HDAC4 expression was not detectable, and this is in agreement with a previous report (20). Thus, expression of HDACs differed between T cells and monocytes under nutrient limitation. However, the role of HDAC4 in the induction of HIV expression during nutrient limitation remains to be determined.

### Increased HIV transcription through GCN2-ATF4 signaling during amino acid deprivation.

**FIG 5:**
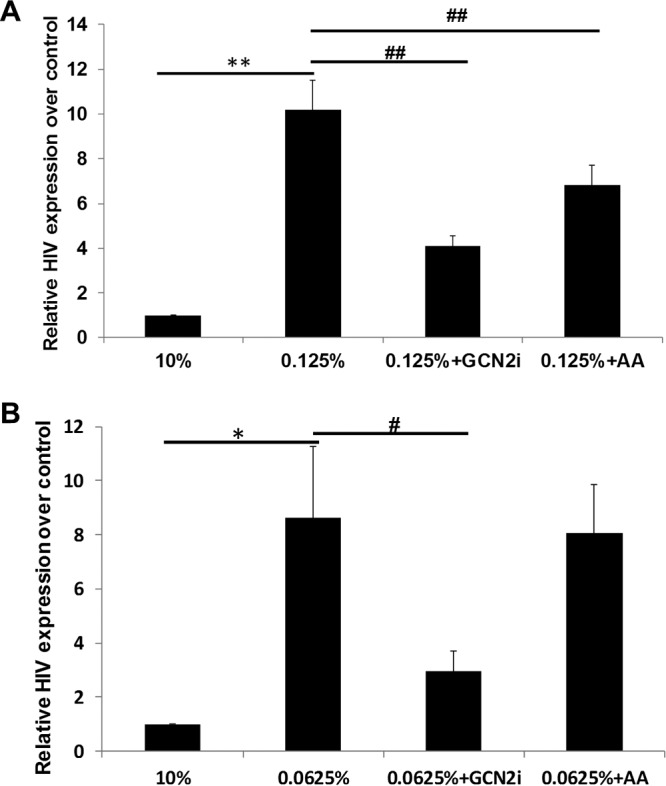
Suppression of GCN2 or amino acid supplementation inhibits induction of HIV expression. J-Lat A1 (A) or U1 (B) cells were nutrient deprived in medium with low levels of serum (0.125% of FBS in medium) in the presence of 10 μM GCN2 inhibitor (SP600125) or 1 mM amino acids (Phe/Lys) for 48 h, and HIV expression was evaluated by qPCR. *, *P* < 0.05; **, *P* < 0.01, compared with 10% FBS medium control; #, *P* < 0.05; ##, *P* < 0.01, compared with nutrient deprivation (0.125% [A] and 0.062% [B]). Experiments were performed a minimum of three times.

To investigate the role of nutrient deprivation-associated GCN2-ATF4 signaling for the induction of HIV transcription, the J-Lat A1 cells were treated with GCN2-specific inhibitor during nutrient deprivation. We found that SP600125, a potent GCN2 inhibitor, effectively suppressed nutrient deprivation-induced HIV transcription in J-Lat A1 cells and HIV reactivation in U1 cells (Fig. 5A and B) (21). Supplementation of the nutrient-deficient cultures of J-Lat A1 cells with amino acids (phenylalanine and lysine) suppressed the activation of HIV transcription (Fig. 5A). However, amino acid supplementation of the nutrient-deprived U1 cells was not effective in suppressing HIV LTR-driven transcription (Fig. 5B). It is possible that HIV expression during nutrient deprivation might engage mechanisms other than the GCN2-ATF4 signaling. Our data suggest that GCN2-ATF4 signaling is involved in the amino acid deprivation-induced HIV activation in immune cells.

### Amino acid deprivation promotes ATF4 binding to HIV LTR.

**FIG 6:**
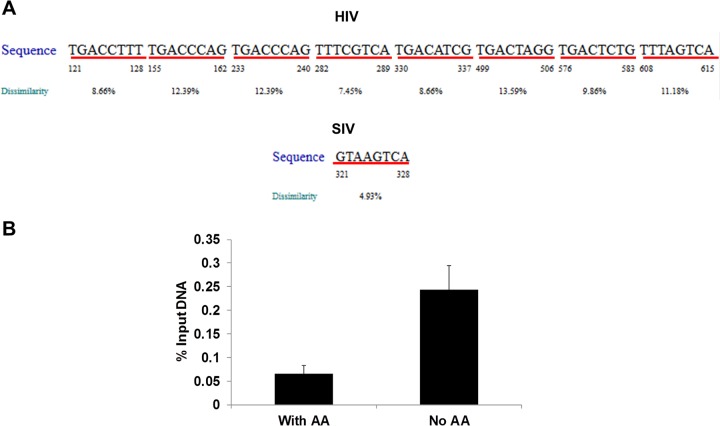
ATF4 is recruited to HIV LTR following amino acid deficiency. (A) Potential CREB/ATF binding sites identified in the HIV (top) and SIV LTR (bottom) region using PROMO software. The underlined sequence is the potential ATF4 binding site in the viral LTR. (B) ATF4 is recruited to HIV LTR after amino acid depletion. J-Lat cells were cultured in medium without amino acids (AA) for 18 h, and ChIP assays were performed with anti-ATF4 antibodies and analyzed by SYBR-qPCR with PCR primers targeting HIV LTR. Fold change of ATF4 binding to HIV LTR was calculated after being normalized to control treatment. *, *P* < 0.05. Experiments were performed three times.

Amino acid deprivation regulates gene expression at multiple steps, including chromatin remodeling, mRNA splicing, RNA export, and translation (22). During amino acid deprivation, a few genes are expressed at higher levels, including that of ATF4. The ATF4 protein can increase gene transcription by binding to the CCAAT-enhancer binding protein-activating transcription factor response elements (C/EBP-ATF) in the target gene promoter. Previously, two C/EBP-ATF binding sites (5′ TGACGTAA 3′) were identified in HIV LTR and confirmed using super-electrophoretic mobility shift assays (EMSAs) *in vitro* (23). The nucleotide sequence analysis of the HIV LTR sequence using PROMO software identified several transcriptional factor binding sites in HIV LTR, including those known for enhancing HIV replication, such as NF-κB, Sp1, NFAT, and YY1 (data not shown). Importantly, we identified several additional potential C/EBP-ATF binding sites in HIV LTR and one C/EBP-ATF binding site in SIV LTR (Fig. 6A). If ATF4 is essential for induction of HIV transcription during amino acid deprivation, ATF4 should be recruited to the HIV promoter to facilitate the viral transcription. To test this hypothesis, chromatin immunoprecipitation (ChIP) assays were performed using anti-ATF4 antibodies following cross-linking of HIV-infected cells in the presence or absence of amino acid deprivation. The PCR primers were targeted to the LTR region of the HIV promoter (−116 bp to +4 bp) (24). Our data show that amino acid deprivation induced significant levels of ATF4 binding to the HIV LTR compared to control cells maintained with amino acid supplementation (Fig. 6B). Our findings support the model in which amino acid deprivation induces ATF4 and increases ATF4 binding to C/EBP-ATF sites in the HIV LTR. This leads to enhanced HIV transcription and replication.

### Induction of ATF4 in T cells reactivates latent HIV.

**FIG 7:**
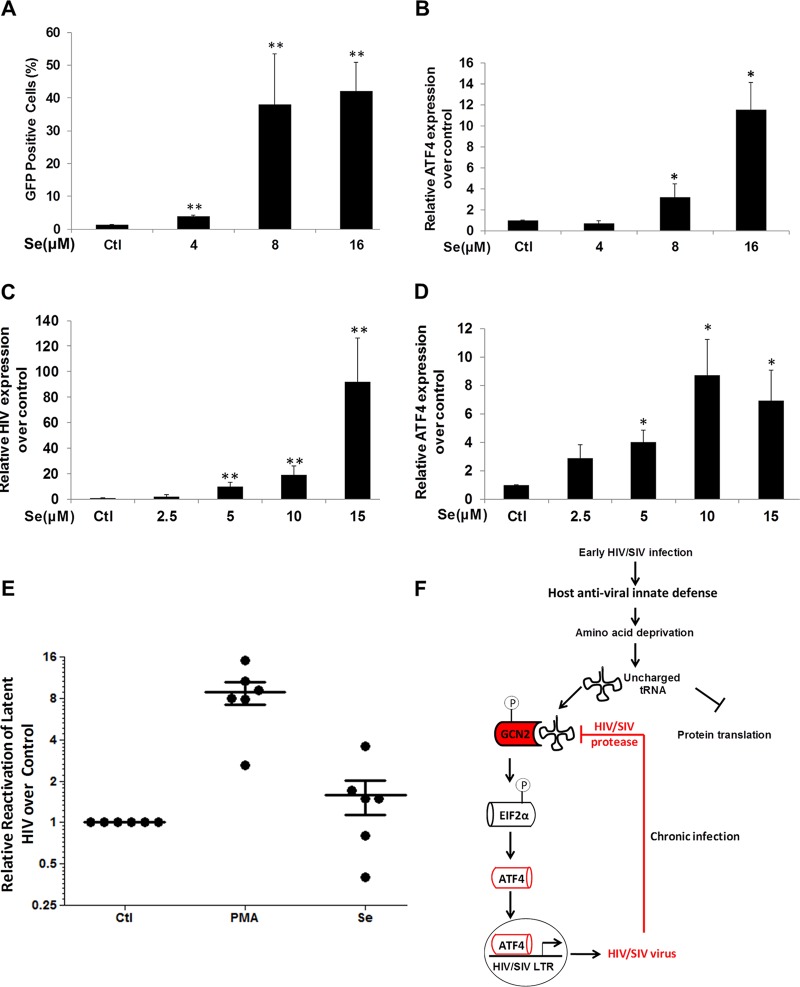
Induction of ATF4 expression by selenium reactivates latent HIV. (A) J-Lat cells were treated with 4 to 16 μM selenium for 24 h, and GFP expression was assessed using flow cytometry to determine latent HIV LTR reactivation. **, *P* < 0.01. (B) J-Lat cells were treated with 4 to 16 μM selenium for 24 h, and ATF4 gene expression was determined by RT-qPCR. *, *P* < 0.05. (C) U1 cells were treated with 2.5 to 15 μM selenium for 24 h, and viral expression was assessed by RT-qPCR to determine latent HIV reactivation. **, *P* < 0.01. (D) The U1 cells were treated with 2.5 to 15 μM selenium for 24 h, and ATF4 gene expression was determined by RT-qPCR. *, *P* < 0.05. (E) Isolated primary CD4^+^ T cells from HIV-infected individuals on suppressive ART were treated with DMSO, 200 ng/ml PMA plus 2 µM ionomycin, or 16 μM selenium for 6 h. Induction of HIV transcription was measured by RT-qPCR for the 5′ LTR region of the virus. (F) The proposed model for the GCN2-eIF2α signaling in early HIV replication. HIV/SIV infection induces antiviral host innate defense to restrict viral production by reducing amino acid deficiency and shutting down protein synthesis. This triggers GCN2-eIF2α signaling and induces ATF4 expression. The virus hijacks this mechanism to recruit ATF4 to the viral LTR and enhance viral replication. During the advanced HIV infection, GCN2 is degraded by HIV protease, the level of ATF4 decreases, and its impact is diminished. Thus, ATF4 may be critical in the initial stages of HIV replication. Data from panels A to D were from three independent experiments.

We sought to examine whether ATF4 is directly linked to the induction of HIV expression in immune cells during nutrient deprivation. The CD4^+^ T cells (J-Lat A1) were treated with selenium. It has been previously reported that selenium induces expression of ATF4 in the Jurkat CD4^+^ T cell line (25). Treatment of J-Lat cells with selenium induced reactivation of HIV and expression of ATF4 (Fig. 7A and B). Similarly, selenium treatment of monocytic U1 cells also induced reactivation of latent HIV and expression of ATF4 (Fig. 7C and D). We expanded this investigation to the primary CD4^+^ T cells from HIV-infected patients receiving long-term antiretroviral therapy (ART). Viral loads in peripheral blood of these patients were below 20 copies/ml. Treatment of primary CD4^+^ T cells with selenium resulted in the induction of HIV expression in cells from 4 of 6 patients (Fig. 7E). In conclusion, activation of ATF4 expression was linked to the induction of HIV expression. Our findings suggest that ATF4 signaling may serve as a new target for limiting early establishment of HIV infection and latency.

## DISCUSSION

Viruses have evolved to overcome antiviral mechanisms employed by mammalian hosts to prevent or eradicate viral infection. Retroviruses are known to mutate their genomes to escape virus-specific host immune response. They also encode accessory viral proteins that dampen the host immune system and/or modulate host cell targets for enhancing viral replication. In recent times, viral strategies have been identified that enable viruses to exploit host antiviral innate responses for establishing viral infection and its persistence (26). Our findings demonstrate that HIV may exploit the host antiviral integrated stress response from the infected host for establishing early stages of viral replication and dissemination.

Gut-associated lymphoid tissue plays an important role in maintaining persistence of HIV reservoirs and chronic immune activation. Due to the frequent exposures to pathogens and environmental stimuli, gut mucosa has evolved to be highly enriched in innate defense mechanisms to control pathogens as well as to limit gut inflammation (27). Effects of active HIV replication and viral antigens on the impairment of gut immunity and epithelial barrier disruption are well recognized. However, our understanding is limited regarding the mechanisms by which HIV is able to initiate early replication and viral production in an innate defense-enriched gut microenvironment that is hostile to pathogens. Our findings of early suppression (at 60 h post-SIV infection) of protein biosynthesis in the gut mucosa represent the initial host innate response to the acute viral infection. By limiting the amino acid availability and protein synthesis, the host antiviral innate response leads to the restriction of the viral production. This innate defense is effective since the retrovirus production is totally reliant on the supply of amino acids and protein synthetic machinery from the host cell. Therefore, the decrease in the protein synthesis during the initial stages of viral infection is an effective strategy of the host innate sensing and response to the viral infection. In contrast, decreased protein synthesis during chronic SIV infection may be attributed to several mechanisms. At 10 weeks post-SIV infection and thereafter, viral loads in the gut mucosa are accompanied by severe CD4^+^ T cell depletion, increased CD8^+^ T cell prevalence, disruption of the gut epithelial barrier integrity, and inflammatory cytokines. Thus, nutritional complications and tissue dysfunction may also contribute to changes in protein biosynthesis during chronic viral infection. Also, at this time, inflammatory cytokines induce cell activation and prime the cellular targets for viral infection and depletion in the gut mucosa. In addition, several other transcription factors may also be readily available in the activated cells to support the viral infection. Last, the viral protease is fully capable of degrading the GCN2 and destroying this innate host defense. HIV protease was previously shown to cleave GCN2 in the cell culture models (28).

Amino acid deficiency and inhibition of protein biosynthesis have been well reported in HIV-infected adults and children or in SIV-infected rhesus macaques (13, 14, 29, 30). Many of these changes are reversed during highly active ART (HAART) (31). Inhibition of host cellular protein synthetic activity is driven by the activation of GCN2-eIF2α signaling, which induces expression of ATF4. We found that both metabolism of amino acids and protein biosynthesis were decreased in the gut mucosa during early SIV infection at a higher magnitude than in chronic SIV infection. A substantial increase in ATF4 expression was detected during acute SIV infection *in vivo* using quantitative reverse transcription polymerase chain reaction (RT-qPCR). We did not detect significant increases in the mRNA levels of GCN2 and eIF2α in the gut mucosa during early SIV infection. This is not surprising since the activation of GCN2 and eIF2α occurs at the posttranslational level through modification by phosphorylation. Analysis of human CD4^+^ T cell cultures and promonocytic cells under an experimental nutrient deprivation condition showed a significant increase in ATF4 expression that correlated with the transcriptional activation of HIV LTR. These data are in agreement with our findings from the SIV model *in vivo* (Fig. 2). Increased levels of ATF4 during early stages of viral infection can have a direct impact on establishing early viral reservoirs due to its ability to induce viral transcription. However, HIV expression was suppressed in the presence of GCN2 kinase inhibitor or by supplementation of amino acids. Our findings reveal a novel mechanism evolved by HIV to exploit the host antiviral restrictive response for establishing its infection. Several previously published reports support our hypothesis. The amino acid deficiency and decreased protein synthesis have been previously reported in HIV, SIV, and several other viral infections (14, 30). Transfection of the ATF4 gene in a T cell line induced HIV LTR transactivation even in the absence of Tat (19). We examined the ATF4 binding site in the LTR in the context of other known transcription factor binding sites, specifically NF-κB, Sp1, and NFAT. They do not seem to overlap the ATF4 binding site, as their consensus binding sequences are quite different. Further, except for ATF4, these well-known HIV transcriptional factors are not among the proteins that were induced by GCN2-ATF4 signaling. Taken together, our data indicated that amino acid deficiency and inhibition of protein biosynthesis as well as the induction of GCN2-ATF4 signaling mainly occur during acute HIV/SIV infection. However, further investigation is warranted regarding GCN2-ATF4 signaling and its role in HIV/SIV replication during chronic infection.

Epigenetic silencing of HIV is induced by histone deacetylase (HDAC) enzymes through chromatin remodeling and supports the maintenance of latent viral reservoirs. Therefore, HDAC inhibitors have been used to reactivate HIV from latency. It has been reported that amino acid starvation and targeted HDAC4 inhibition resulted in reactivation of latent HIV in a T cell line but not in a promonocytic U1 cell line that lacks HDAC4 (20). We examined HDAC expression and HIV transcription in CD4^+^ T cell cultures and promonocytic U1 cells under conditions of nutrient deprivation. Our data showed that the expression of HDAC1 to HDAC4 was induced and not suppressed in the CD4^+^ T cell cultures. Surprisingly, expression of HDAC1 and HDAC2, and to a lesser extent of HDAC3, was increased in the monocytic cell cultures. However, HDAC4 expression was too low for detection in U1 cells and was not altered under the nutrient deprivation conditions, as previously reported (20). It seems that HDACs may exert a differential effect on transcription of HIV in lymphoid and myeloid cells during the amino acid deprivation.

Previously, ATF was reported to have a potential effect on HIV replication (19). Several potential ATF/CREB binding sites were identified in the HIV LTR region using EMSAs *in vitro* (23, 32). Another study reported that DNA methylation of these ATF/CREB sites was involved in establishing HIV latency (33). ATF4 was recently shown to activate HIV replication *in vitro* by transactivating HIV LTR with as well as without Tat (19). However, the relevance of ATF4 expression during early HIV infection is not defined. In the present study, we found that activation of GCN2-ATF4 was caused by amino acid deprivation, which facilitates ATF4 binding to the HIV LTR to drive HIV transcription/replication. Consequently, inhibition of GCN2 or supplementation of amino acids during the serum starvation suppressed reactivation of latent HIV. Although 4-phenylbutyrate is an effective GCN2-eIF2α-ATF4 signaling inhibitor, it is also a pan-HDAC inhibitor and is known to reactivate latent HIV (34, 35). Therefore, we did not include 4-phenylbutyrate in our assays since the application of 4-phenylbutyrate might complicate our analysis of latent HIV reactivation.

It is interesting that ATF4 expression may have a defining role in establishing early stages of HIV/SIV infection. It may also be utilized for activating latent HIV reservoirs. Epigenetic modification of ATF/CREB binding sites appeared to be essential for HIV reactivation from latency (33). Induction of ATF4 expression led to reactivation of latent HIV *in vitro* and *ex vivo*, suggesting the role of ATF4 in maintaining HIV latency. In agreement with previously reported findings (19), addition of selenium to CD4^+^ T cells and monocytic cells induced expression of ATF4. We found that selenium, as an inducer of the ATF4 expression, also reactivated expression of latent HIV in both J-Lat A1 cells and U1 cells, as well as in primary CD4^+^ T cells from HIV^+^ patients under suppressive ART. The association of selenium with increased HIV viral loads in AIDS patients was reported in several clinical trials. In a randomized trial, micronutrients plus selenium supplementation resulted in higher levels of genital HIV-1 shedding than those with placebo in ART-naive AIDS patients (36). The effect was lost when the same micronutrients were administered without selenium at identical dosages in patients. Instead, administration of micronutrients lacking in selenium resulted in lower levels of HIV RNA viral loads (37). To investigate the effect of selenium on HIV viral loads, a clinical study was performed in 340 HIV-infected pregnant women, which demonstrated that the presence of high levels of selenium resulted in increased risk of genital HIV-1 shedding (38). A recent study showed that selenium supplementation among some of the therapy-naive HIV-infected women led them to show higher levels of viral RNA in their breast milk (39). These findings indicate the role of selenium in supporting HIV expression *in vivo*. We found that selenium could reactivate latent HIV in primary CD4^+^ T cells from some HIV-infected individuals receiving suppressive HAART *ex vivo*. It appears that selenium induced HIV replication before ATF4 expression in CD4^+^ T cells (Fig. 7A and B), indicating that selenium induction of HIV replication may involve additional signaling pathways. Although the effect of selenium in reactivation of latent HIV was modest, it further supported the role of ATF4 in viral activation. In addition, our findings may provide an explanation for increased susceptibility to HIV infection among children and adults suffering from malnutrition in developing countries (40, 41). Induction of the GCN2-ATF4 pathway under the conditions of amino acid starvation may support HIV transcription and spread in these individuals. The role of stress in reactivation of HIV reactivation is underinvestigated (42). Our data indicated that nutrient stress-induced GCN2-ATF4 signaling can be targeted for reactivating latent HIV during HAART.

In summary, our data show that early stages of SIV infection induce inhibition of protein synthesis and amino acid depletion in the gut mucosa, potentially an early antiviral host response, through GCN2-ATF4 signaling. Increased expression of ATF4 during this early stage of SIV infection has a direct impact on the viral transcription as a viral LTR transactivator. Our findings suggest a model that the antiviral host response to HIV/SIV infection through GCN2-ATF4 signaling may be exploited by the virus to activate its transcription and expression, may be highly relevant during the early stages of infection *in vivo*, and may serve as an excellent target for prevention of viral dissemination.

## MATERIALS AND METHODS

### Human cell cultures.

J-Lat A1 (J-Lat) cells or U1 cells were obtained from the NIH AIDS Reagent Program and cultured in RPMI 1640 medium with 10% fetal bovine serum (FBS) and 1% penicillin-streptomycin (Pen-Strep) in a 37°C incubator containing 5% CO_2_ (43, 44). The reactivation of HIV LTR was quantified by GFP expression using flow cytometry. The data were analyzed using FlowJo Software for J-Lat cells or by RT-qPCR for both J-Lat and U1 cells.

### Metabolic analysis.

An untargeted primary metabolic analysis of macaque gut tissue was performed by gas chromatography-time of flight (GC-TOF) mass spectrometry (MS). Ten to 20 mg of cryopreserved gut tissue was obtained from SIV-infected animals during the early stage at 60 h postinfection (*n =* 3) or during chronic stage at 10 weeks postinfection (*n =* 4) and from SIV-negative controls (*n =* 4). Tissue samples were homogenized in an extraction mixture of acetonitrile, isopropanol, and water (3:3:2) and centrifuged, and supernatants were evaporated. Dried pellets were dissolved in 50% acetonitrile, evaporated, and subjected to a two-step derivatization using methoximation and trimethylsilylation. The GC-MS analysis was performed using an Agilent 7890 gas chromatography system coupled to an Agilent 5977A mass spectrometer by the West Coast Metabolomics Center at UC Davis, as previously described (45). Overrepresentation analysis (ORA) was performed in Metabolites Set Enrichment Analysis (MSEA) software (http://www.metaboanalyst.ca) using the metabolic pathways library from MSEA for all metabolites showing a 1.3-fold change.

### Serum or amino acid starvation/adding assays.

After being washed twice with phosphate-buffered saline (PBS), 1 × 10^6^ J-Lat A1 cells or U1 cells were cultured in a 12-well plate in RPMI (US Biological, Salem, MA) with serum (Invitrogen), without serum, or without amino acids for 24 h to 72 h. Cells were collected for either flow cytometry or RNA extraction. Also, 10 μM GCN2 kinase inhibitor SP600125 or 1 mM amino acid was added to the J-Lat A1 or U1 cell cultures at low levels of serum.

### Primary CD4^+^ T cell isolation and treatments.

Peripheral blood samples were collected from healthy donors (*n =* 4) or from HIV-infected individuals receiving suppressive antiretroviral therapy (ART) for >3 years (*n =* 6). The CD4^+^ T cell counts in peripheral blood samples ranged from 264 to 1,100 cells/mm^3^, and plasma viral loads were <20 copies per ml as measured by qPCR. CD4^+^ T cells were isolated using the EasySep kit (StemCell Technologies Inc., Vancouver, BC, Canada) as previously described (44, 46). The purified CD4^+^ T cells were plated at a density of 1 × 10^6^ cells and treated with dimethyl sulfoxide (DMSO), 200 ng/ml phorbol myristate acetate (PMA) plus 2 µM ionomycin, or 16 µM selenium for 6 h, and the cell pellets were collected for RNA isolation.

### Rhesus macaques and viral infections.

Rhesus macaques (*Macaca mulatta*) were obtained from the California National Primate Research Center (CNPRC). Peripheral blood and intestinal tissue samples were obtained from 21 rhesus macaques (age ranging from 4 to 6 years). Fifteen animals were intravenously infected with 1,000 50% tissue culture infective doses (TCID_50_) of SIVmac251 and necropsied at either 60 h (*n* = 3), 2 weeks (*n* = 5), 10 weeks (*n* = 4), or 26 weeks (*n* = 3) following SIV infection. Six healthy SIV-negative rhesus macaques served as negative controls. Immunological and virological findings from some of these animals were previously described (17, 18). At 60 h post-SIV infection, there is a low level of viral infection in the gut and peripheral blood, prior to any detectable loss of CD4^+^ T cells due to infection, while at 1 to 2 weeks post-SIV infection, there is a wider dissemination of viral infection to the susceptible cellular targets that is accompanied by the loss of mucosal CD4^+^ T cells. At 8 weeks onward following SIV infection, the viral loads in the peripheral blood reach a steady state. Therefore, time points thereafter represent the chronic stage of viral infection, which includes both 10- and 26-week infection time points in our study. Severe CD4^+^ T cell depletion and viral persistence are detected in the gut mucosa in the chronic SIV infection.

### Rhesus macaque gene expression by DNA microarray analysis and real-time PCR.

RNA was isolated from gut tissue samples using the RNeasy kit (Qiagen, Valencia, CA). mRNA amplification, labeling, hybridization to GeneChips (Affymetrix), staining, scanning, and statistical analysis of the data were performed as described previously (18). Fluorescence intensity values from scanned GeneChips were subjected to comparative analyses utilizing R statistical software and BRB Array Tools and Ingenuity Pathway Analysis (IPA) software as previously described (18). The previously analyzed data files were mined for the expression of GCN2-ATF4 signaling-related genes.

For the validation of the DNA microarray-based gene expression data, rhesus macaque-specific primers/probes for GCN2, eIF2α, and ATF4 genes were obtained and real-time PCR was performed to quantitate RNA levels of these genes in the gut tissue samples of SIV-infected animals and negative controls.

### ChIP.

The chromatin immunoprecipitation (ChIP) assay was performed as previously described (44, 46, 47). Briefly, J-Lat A1 cells were fixed in 1% formaldehyde and suspended in lysis buffer containing 1% SDS, 10 mM EDTA, 50 mM Tris-HCl, pH 8.1 (ChIP assay kit; Millipore), and a protease inhibitor cocktail (Sigma-Aldrich). Lysates were sonicated to obtain DNA fragments of 200 to 1,500 bp. After incubation with anti-ATF4 antibodies (ABE387; EMD-Millipore) overnight, the immune complex was retrieved by incubating lysates for 45 min with 50 µl of protein A/G agarose beads saturated with bovine serum albumin (BSA)-salmon sperm DNA. Following the washes, the chromatin was eluted and reverse cross-linked overnight. DNA was extracted (Qiagen PCR purification kit), and quantitative real-time PCR was performed using Agilent Brilliant Ultra-Fast SYBR green QPCR reagent and the 7500 real-time PCR system. The upstream primer sequence was 5′ AGCTTGCTACAAGGGACTTTCC 3′, and the downstream primer sequence was 5′ ACCCAGTACAGGCAAAAAGCAG 3′. These PCR primers targeted the HIV LTR region (−116 bp to +4 bp).

### HIV gene expression by real-time PCR analysis.

Total RNA was isolated using the RNeasy kit (Qiagen) followed by digestion with DNase I (Invitrogen). First-strand cDNA was synthesized using Superscript III (Invitrogen). Real-time PCR (TaqMan) was performed on a ViiA7 detector using a primer/probe set as previously reported (44, 46). The glyceraldehyde-3-phosphate dehydrogenase (GAPDH) primer/probe set was purchased from Applied Biosystems and used for control PCR.

### HIV infection of primary CD4^+^ T cells.

Primary CD4^+^ T cells were isolated from peripheral blood samples of healthy HIV-negative donors (*n* = 4) and infected with HIV-1 (HIV-1 IIIB expanded in Jurkat T cells, 100 to 200 ng p24-gag) by spinoculation as described previously (48). Following an exposure to HIV for 24 h, cells were washed with PBS, cell supernatants were collected after 3 to 5 days, and HIV p24 levels were measured by enzyme-linked immunosorbent assay (ELISA). The cells were also collected for RNA extraction and viral RNA measurements by RT-qPCR.

### HIV RNA quantification in patient samples.

Total RNA was extracted with the Qiagen RNeasy kit, including a DNA digestion step. Quantitative RT-PCR was performed using TaqMan Fast Virus 1-Step master mix (Applied Biosystems) in a ViiA7 thermocycler. Transcripts containing the 3′ poly(A) region were amplified as previously reported (44).

### Ethics statement.

This study was carried out under the recommendations of the Public Health Services Policy on Humane Care and Use of Laboratory Animals. Animals were housed at the California National Primate Research Center, and procedures were performed according to a protocol approved by the Institutional Animal Care and Use Committee of the University of California, Davis. Appropriate sedatives, anesthetics, and analgesics were used during handling and surgical manipulations to ensure minimal pain, suffering, and distress to animals. Animals were euthanized in accordance with the American Veterinary Medical Association (AVMA) Guidelines for the Euthanasia of Animals. Human samples were obtained under informed written consent and a protocol approved by the UC Davis Institutional Review Board (IRB 219139).

### Statistical analysis.

Means and standard errors (SEs) were calculated for all data points from at least 3 independent experiments. Statistical significance was determined using the two-way Student *t* test, where a *P* value of <0.05 was considered significant.

### Data availability.

The original microarray data analysis was previously reported (18).
